# Intracellular and Extracellular Metabolites from the Cyanobacterium *Chlorogloeopsis fritschii,* PCC 6912, During 48 Hours of UV-B Exposure

**DOI:** 10.3390/metabo9040074

**Published:** 2019-04-16

**Authors:** Bethan Kultschar, Ed Dudley, Steve Wilson, Carole A. Llewellyn

**Affiliations:** 1 Department of Biosciences, Swansea University, Singleton Park, Swansea SA2 8PP, UK; 2 Swansea University Medical School, Swansea University, Singleton Park, Swansea SA2 8PP, UK; E.Dudley@swansea.ac.uk; 3 Unilever Corporate Research, Colworth Park, Sharnbrook, Bedfordshire MK44 1LQ, UK; Steve.Wilson@Unilever.com

**Keywords:** cyanobacteria, *C. fritschii*, UV-B, PAR, time-series, intracellular, extracellular, metabolites, GC–MS

## Abstract

Cyanobacteria have many defence strategies to overcome harmful ultraviolet (UV) stress including the production of secondary metabolites. Metabolomics can be used to investigate this altered metabolism via targeted and untargeted techniques. In this study we assessed the changes in the intra- and extracellular low molecular weight metabolite levels of *Chlorogloeopsis fritschii* (*C. fritschii*) during 48 h of photosynthetically active radiation (PAR) supplemented with UV-B (15 µmol m^−2^ s^−1^ of PAR plus 3 µmol m^−2^ s^−1^ of UV-B) and intracellular levels during 48 h of PAR only (15 µmol m^−2^ s^−1^) with sampling points at 0, 2, 6, 12, 24 and 48 h. Gas chromatography–mass spectrometry (GC–MS) was used as a metabolite profiling tool to investigate the global changes in metabolite levels. The UV-B time series experiment showed an overall significant reduction in intracellular metabolites involved with carbon and nitrogen metabolism such as the amino acids tyrosine and phenylalanine which have a role in secondary metabolite production. Significant accumulation of proline was observed with a potential role in stress mitigation as seen in other photosynthetic organisms. 12 commonly identified metabolites were measured in both UV-B exposed (PAR + UV-B) and PAR only experiments with differences in significance observed. Extracellular metabolites (PAR + UV-B) showed accumulation of sugars as seen in other cyanobacterial species as a stress response to UV-B. In conclusion, a snapshot of the metabolome of *C. fritschii* was measured. Little work has been undertaken on *C. fritschii*, a novel candidate for use in industrial biotechnology, with, to our knowledge, no previous literature on combined intra- and extracellular analysis during a UV-B treatment time-series. This study is important to build on experimental data already available for cyanobacteria and other photosynthetic organisms exposed to UV-B.

## 1. Introduction

Cyanobacteria are gram-negative bacteria with the ability to photosynthesise, assimilating CO_2_ into a variety of biochemical compounds through different metabolic pathways [1]. Cyanobacteria can thrive in a wide variety of extreme habitats such as high ultraviolet radiation (UVR) due to their adaptive capabilities such as the production of secondary metabolites [1]. Metabolomics can be used to determine changes at the metabolite level during varying environmental stimuli and is a useful tool in cyanobacterial research [2]. The metabolome provides information closely reflecting the interaction between an organism and its environment. Some metabolites produced by cyanobacteria under stress conditions are unique and are of increasing interest from a biotechnological perspective as sustainable sources of ingredients in a variety of industries [3,4].

The effect of UVR on cyanobacteria has been widely researched including the interaction with biomolecules, production of reactive oxygen species (ROS) which cause oxidative stress, impaired growth, partial inhibition of photosynthesis and decreased enzyme activity [5,6,7]. UVR also has a role as an activator of secondary metabolite production such as mycosporine-like amino acids (MAAs) [8] and other protective secondary metabolites [9]. Many studies have been conducted to identify these targeted intracellular metabolites during UV-B and UV-A exposure in *Lyngbya* sp. CU2555 [10], *Nostoc commune* [11], *Anabaena variabilis* PCC 7937 [12], *Calothrix* sp. [13] and *Chlorogloeopsis fritschii* (*C. fritschii)*, PCC 6912, [14] to name a few. Other studies conducted have sought to evaluate changes at the protein level [15,16], targeted and untargeted metabolomic analysis using different intensities of UV-B [17] and combined metabolomic and proteomic analysis during UV-A exposure [18].

Cyanobacteria convert CO_2_ into reduced carbon which forms the backbone of metabolites and are central to life. Like many other microorganisms, cyanobacteria release these carbon-based primary and secondary metabolites into their surrounding area which drives carbon cycling within microbial communities [19,20]. These released metabolites are by-products of metabolism within cells and make up a small proportion of the dissolved organic matter (DOM) pool within freshwater and marine ecosystems [19]. Consisting of a variety of chemical compositions such as; polysaccharides, proteins, lipids, organic compounds or inorganic molecules, they are released for communication, structural organisation, and defence against biotic and abiotic factors [20,21,22]. The uptake and release of metabolites change with varying environments; examples include the release of exopolysaccharides during high light and UVR [11,23].

Monitoring industrially relevant metabolites released by microorganisms into their surroundings is a widely used technique in the fermentation industry. It can be used in bioprocess monitoring, fermentation biomarker identification, for monitoring metabolite levels in fermentation processes and microbial contamination [24,25].

Combining intracellular and extracellular analysis is useful in the study of cyanobacteria providing a more holistic picture of metabolite production during growth and its response to different environmental conditions [25,26].

Little work has been undertaken on monitoring both intracellular and extracellular metabolites in cyanobacteria especially *C. fritschii,* a potential candidate for use in industrial biotechnology due to its scalability [27] and tolerance to different growth conditions [28,29,30]. In this study, we observe the changes in metabolites produced by *C. fritschii* during 48 h of UV-B exposure as detected by untargeted gas chromatography-mass spectrometry (GC–MS). We were able to identify metabolites with altered levels comparing UV-B treatment (PAR + UV-B) to cultures irradiated with PAR only.

## 2. Results

### 2.1. Intracellular and Extracellular Analysis of C. fritschii during UV-B Stress Response

The metabolite profiles of *C. fritschii* cultures (*n* = 3) were investigated during 48 h of UV-B exposure. At each time point (0, 2, 6, 12, 24 and 48 h) intracellular and extracellular metabolites were analysed by untargeted GC–MS to evaluate the global changes in metabolite production during UV-B stress.

A total of 300 and 412 peaks were detected from the intracellular and extracellular time-series data respectively (Tables S1 and S2). Using a match factor of 60% or above, 135 and 218 peaks were putatively identified within the intracellular and extracellular data respectively (Tables S1 and S2). The identified chemical structures belonged to a variety of classes such as; acids, alcohols, amino acids, aromatics, fatty acids, heterocycles and sugars.

**Figure 1:**
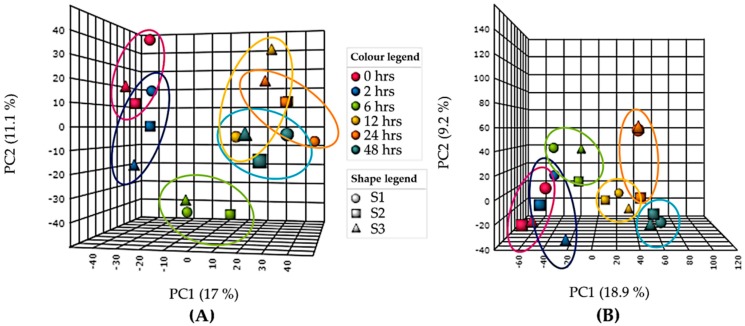
Principle component analysis (PCA) of (**A**) intracellular and (**B**) extracellular gas chromatography-mass spectrometry (GC–MS) data of UV-B exposed (PAR + UV-B) *Chlorogloeopsis fritschii* (*C. fritschii*) cultures showing PC1 vs PC2 only. Each ring represents distribution of biological replicates. S1 = replicate 1, S2 = replicate 2, S3 = replicate 3.

A Principle Component Analysis (PCA) model was used as an unsupervised multivariate statistical tool to plot and visualise the variance between UV-B exposed samples over time. A total variance of 37.2% for intracellular (Figure 1A, PC1 = 17%, PC2 = 11.1%, PC3 = 9.1%) and 36.4% for extracellular (Figure 1B, PC1 = 18.9%, PC2 = 9.2%, PC3 = 8.3%) was observed.

The results of the PCA for intracellular samples (Figure 1A) showed good separation over time between the control (0 h) and 6, 12, 24 and 48 h of UV-B. Less variation was observed between 0 and 2 h of UV-B with clustering seen between 12, 24 and 48 h of UV-B. This result was consistent with the two sample T-test results comparing control (0 h) with each time point where the number of significant features increases with length of UV-B exposure (Table S1). After a one-way analysis of variance (ANOVA) with repeated measures, 112 statistically significant peaks were observed with *p* ≤ 0.05 (Figure S1A), 10 of which remained significant after Bonferroni correction.

From the extracellular data PCA (Figure 1B) a similar pattern was observed with increasing variance with increasing length of UV-B exposure. Statistically significant changes between control (0 h) and each time point, measured using a two-sample T-test also showed increasing significance (*p* ≤ 0.05) with increasing length of UV-B up to 24 h (Table S2). A one-way ANOVA with repeated measures calculated 114 statistically significant peaks with *p* ≤ 0.05 (Figure S1B).

#### 2.1.1. Intracellular Metabolites

**Figure 2:**
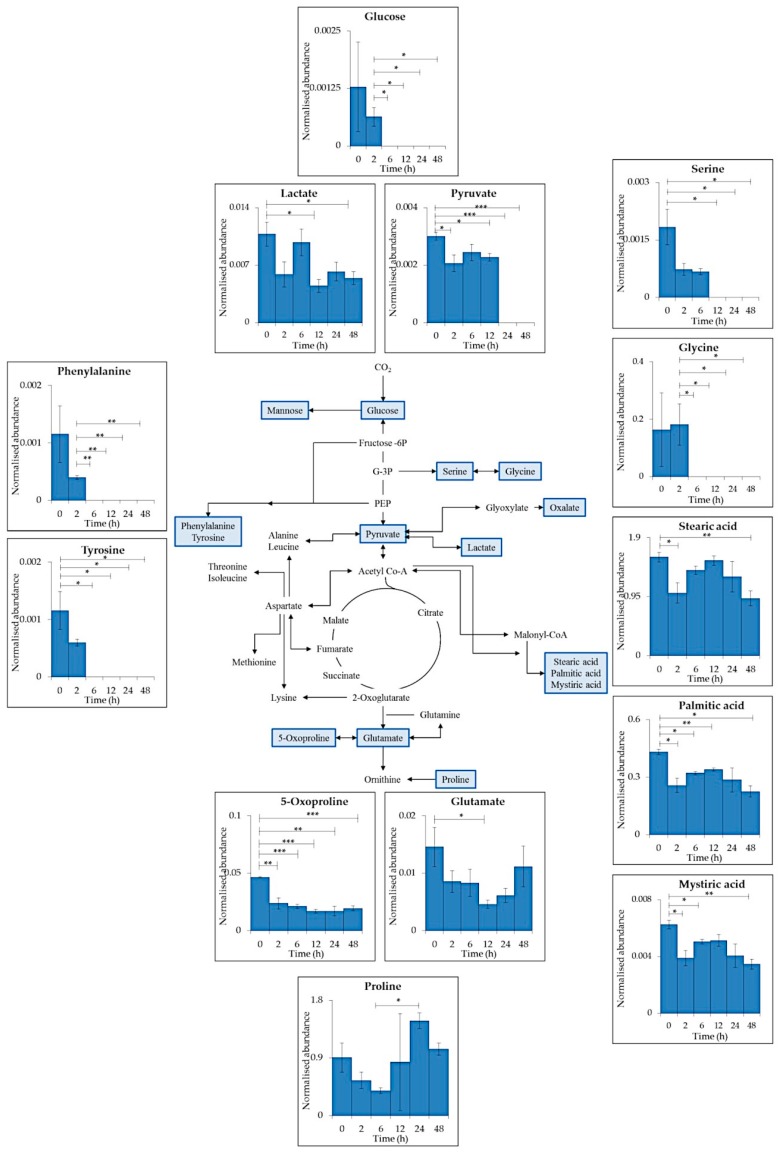
Schematic representation of a generalised reduced carbon metabolism in the cyanobacterium *C. fritschii* showing glycolysis, the citric acid (TCA) cycle, amino acid and fatty acid biosynthesis. Primary metabolites identified in intracellular samples using GC–MS are highlighted in blue with each insert presenting mean values of normalised abundance (normalised to internal standard and dry weight) ± standard error of each metabolite during supplemented UV-B exposure PAR + UV-B). Statistical significance between control (0 h) and UV-B exposure (2, 6, 12, 24 and 48 h) and between each treatment time point was measured using a two-sample T-test with equal variance; * = 0.05 ≥ *p* ≥ 0.01, ** = 0.01 ≥ *p* ≥ 0.001 and *** = *p* ≤ 0.001.

28 metabolites (13 represented for simplicity, Figure 2), selected as being involved in the central carbon and nitrogen metabolism within cyanobacteria, were identified within the intracellular GC–MS results Table S3). Many changes in metabolite levels were observed comparing between time points. Glucose, pyruvate and lactate all decreased in abundance after UV-B exposure with significant reduction after 2 h (pyruvate *p* ≤ 0.05, 0 vs. 2 h), 6 h (glucose *p* ≤ 0.05, 2 vs. 6 h) and 12 h (lactate *p* ≤ 0.05, 0 vs. 12 h). Lactate was present during the whole time course whereas glucose and pyruvate were below detection limit after 6 h (*p* ≤ 0.05) and 24 h (*p* ≤ 0.001) respectively.

6 proteinogenic amino acids were detected; serine (ser), glycine (gly), glutamate (glu), proline (pro), tyrosine (tyr) and phenylalanine (phe). All detected amino acids decreased after 6 or 12 h of exposure with the exception of pro. A decrease in tyr, phe and gly was seen after 6 h (tyr *p* ≤ 0.05, 0 vs. 6 h; phe *p* ≤ 0.01, 2 vs. 6 h; gly *p* ≤ 0.05, 2 vs. 6 h) of UV-B followed by no detection at 6, 12, 24, and 48 h (tyr *p* ≤ 0.05; phe *p* ≤ 0.01; gly *p* ≤ 0.05). Ser and glu decreased significantly after 12 h of treatment (ser *p* ≤ 0.05, 0 vs. 12 h; glu *p* ≤ 0.05, 0 vs. 12 h), ser was below detection limit between 12 and 48 h (*p* ≤ 0.05) whereas glu was detected throughout the time series. Proline showed no significant decrease after UV-B exposure with a significant increase observed after 24 h (*p* ≤ 0.05, 6 vs. 24 h).

The fatty acids stearic acid, palmitic acid and mystiric acid all decreased significantly after 2 h of treatment (*p* ≤ 0.05) and their abundance remained lowered throughout the time series (stearic acid *p* ≤ 0.01, 0 vs. 48 h; palmitic acid *p* ≤ 0.05, 0 vs. 48 h; mystiric acid *p* ≤ 0.01, 0 vs. 48 h).

##### Carotenoid and MAA Analysis

**Figure 3:**
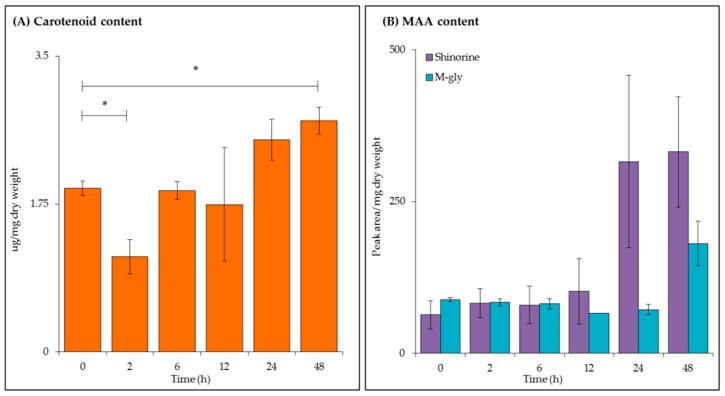
Carotenoid and mycosporine-like amino acid (MAA) analysis of *C. fritschii* extracts during UV-B exposure. **(A)** Total carotenoid concentration as measured by UV-visible spectroscopy and **(B)** shinorine and mycosporine-glycine (m-gly) content measured by high-performance liquid chromatography (HPLC) analysis. All values are the mean of three biological replicates (normalised to dry weight) ± standard error. Statistical significance was measured using a two-sample T-test with equal variance; * = 0.05 ≥ *p* ≥ 0.01, ** = 0.01 ≥ *p* ≥ 0.001 and *** = *p* ≤ 0.001.

Carotenoid concentration (Figure 3A) and MAA content (Figure 3B) were analysed by UV-visible spectroscopy and high performance liquid chromatography (HPLC) respectively. Total carotenoid concentration decreased after 2 h (*p ≤* 0.05); with a steady significant increase up to 48 h with a final concentration of 2.59 μg/mg dry weight (*p ≤* 0.05).

As described above, UV-B also induces the production of the photoprotective compounds, MAAs. The two forms found in *C. fritschii* are mycosporine-glycine (m-gly) and shinorine [29], both were detected during this experiment with peaks identified using their retention time and absorption maxima (λmax) values. As expected an increase in shinorine (retention time ~4.9 min, λmax = 334 nm) was observed with increasing length of UV-B exposure. No significance was observed with m-gly (retention time ~10.8 min, λmax = 310 nm) content during this experimental time series.

#### 2.1.2. Extracellular Metabolites

**Figure 4:**
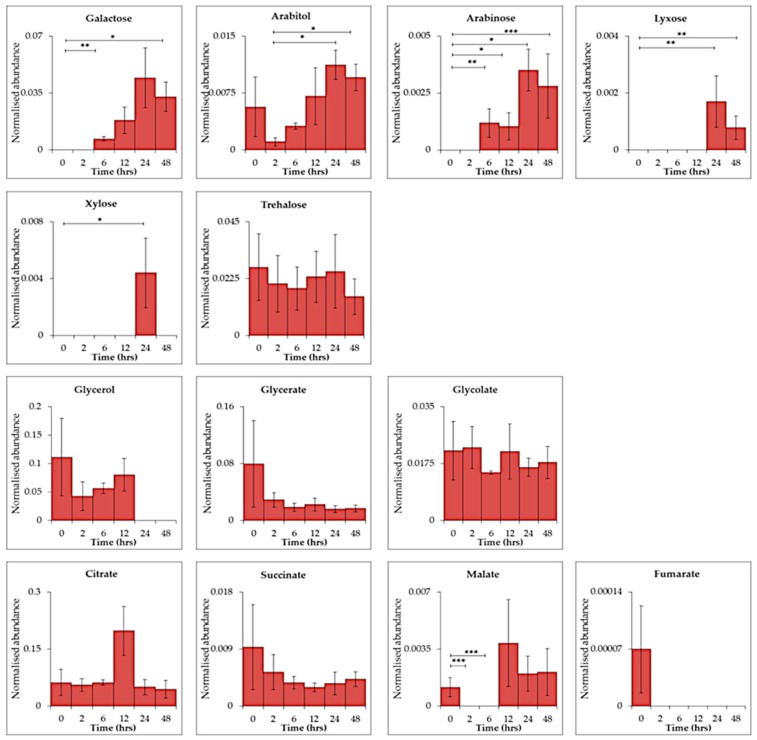
Time-series exometabolomics data of *C. fritschii* over 48 h of UV-B exposure showing primary metabolites found in extracellular samples only. Statistical significance was measured using a two-sample T-test comparing control (0 h) and UV-B exposure (2, 6, 12, 24 and 48 h) and between each treatment time point, * = 0.05 ≥ *p ≥* 0.01, ** = 0.01 ≥ *p ≥* 0.001 and *** = *p ≤* 0.001.

29 biologically relevant dissolved metabolites (Table S3) were detected within the extracellular data set (13 represented for simplicity, Figure 4). Citrate, a component of BG-11 medium [31] and involved in the citric acid (TCA) cycle, was consistently detected throughout the time series (ANOVA, *p ≤* 0.05) along with succinate. Other TCA substrates; malate and fumarate were also detected at 0 h with decreasing abundance after 2 h of UV-B (malate, *p ≤* 0.001). Other metabolites detected at 0 h which decreased after UV-B exposure were leucine (*p ≤* 0.01, 0 vs. 6 h), putrescine (*p ≤* 0.05, 0 vs. 2 h) octanoic acid (*p ≤* 0.001, 0 vs. 24 h) mystiric acid (*p ≤* 0.01, 2 vs. 24 h) and fructose (*p ≤* 0.01, 0 vs. 2 h). Accumulation of the sugars galactose, xylose, lyxose and arabinose was seen after 6 h (galactose *p ≤* 0.01; arabinose *p ≤* 0.01) 12 h (arabinose *p ≤* 0.05), 24 h (arabinose *p ≤* 0.05; xylose *p ≤* 0.05; lyxose *p ≤* 0.01) and 48 h (galactose *p ≤* 0.05; arabinose *p ≤* 0.001; lyxose *p ≤* 0.01) of UV-B exposure. Trehalose was also identified throughout the time series with no significant changes.

### 2.2. Intracellular Analysis of C. fritschii (PAR Only)

#### Intracellular Metabolites

**Figure 5:**
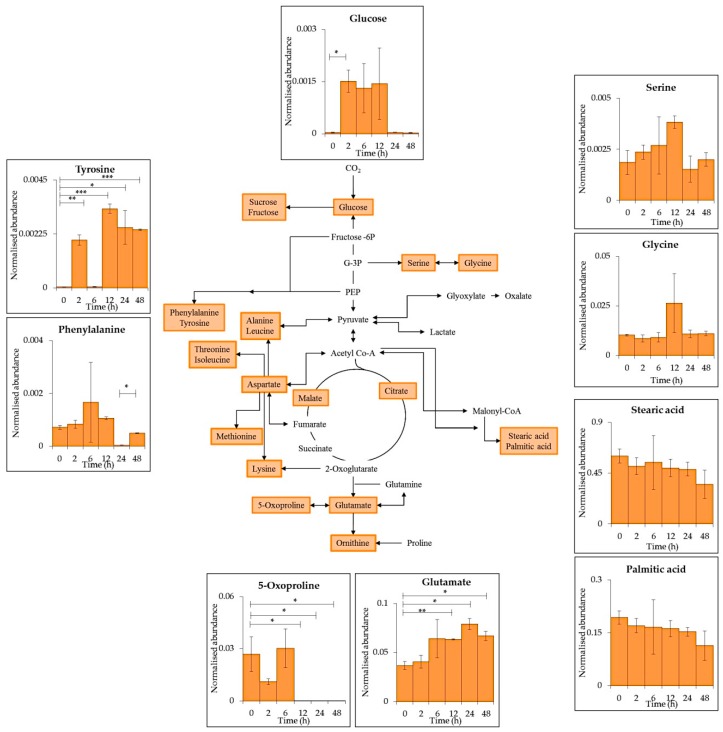
Schematic representation of a generalised reduced carbon metabolism in the cyanobacterium *C. fritschii* showing glycolysis, TCA cycle, amino acid and fatty acid biosynthesis. Primary metabolites identified in intracellular samples using GC–MS are highlighted in orange with each insert presenting mean values of normalised abundance (normalised to internal standard and dry weight) ± standard error of common metabolites found during PAR only conditions and supplemented UV-B (Figure 2). Statistical significance between 0 h and each time point as well as between time points were measured using a two-sample T-test with equal variance; * = 0.05 ≥ *p ≥* 0.01, ** = 0.01 ≥ *p ≥* 0.001 and *** = *p ≤* 0.001.

A time-series analysis of PAR only (without UV-B supplementation) over 48 h (Table S4) revealed 35 key primary intracellular metabolites (Table S3). 12 were commonly identified between PAR only conditions and during UV-B supplementation (nine represented for simplicity in pathway schematic, Figure 5).

**Figure 6:**
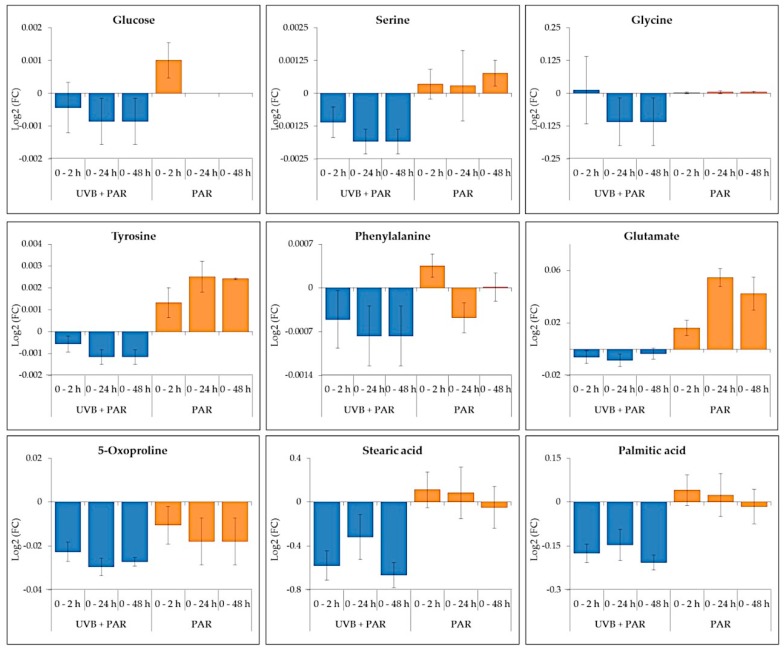
The changes in common metabolites identified in both UV-B + PAR and PAR only experiments comparing 0 h with 2, 24 and 48 h. Data are presented as Log2 Fold Change (FC) ± standard error.

In general, comparing both supplement UV-B and PAR only, the nine common metabolites (Figure 6) showed differences in log2 fold change (FC). The metabolites detected during supplemented UV-B showed negative log2(FC) values which corresponds to reduced metabolite abundances compared to 0 h. Positive log2 (FC)) was generally observed for metabolites detected during PAR only indicating increased abundances compared to 0 h. The main exception is 5-oxoproline that reduced in both UV-B + PAR and PAR only experiments.

A significant accumulation of glu (*p* ≤ 0.01) was observed after 12 h of PAR only whereas a significant decrease was seen after 12 h of UV-B exposure. Tyr accumulation (*p* ≤ 0.001) was also seen after 2 and 12 h of PAR only conditions with a significant decrease during UV-B treatment. Palmitic and stearic acid remained relatively stable over time with a significant reduction during UV-B exposure observed. Gly and ser abundance remained consistent with significant decreases after 6 and 12 h respectively during UV-B exposure.

## 3. Discussion

### 3.1 Intracellular Metabolite Changes and Pathway Analysis

UV-B exposure is known to reduce growth, photosynthesis and nitrogen fixation in cells to divert energy from key primary pathways to adaptive mechanisms such as; the production of secondary metabolites, MAAs; antioxidant production and DNA/protein repair [32]. A reduction in average dry weight was measured over 24 h of UV-B exposure (Figure S2) with the recovery of the initial biomass concentration and further growth measured between 24 and 48 h. These results showed no significance (*p ≥* 0.05) across the time series suggesting acclimation of cells to UV-B where damage to photosynthetic systems are counterbalanced by repair and mitigation strategies. A reduction in carotenoid concentration (Figure 3A) was observed after 2 h (*p* ≤ 0.05) due to damage to photosystems caused by UV-B. Accumulation of total carotenoids could be indicative of antioxidant activity as a response to ROS production [6,33].

A reduction in glucose, pyruvate and lactate (Figure 2) could indicate a reduction in CO_2_ fixation via photosynthesis and further biochemical processes. This could be due to the reduced production of ATP and NADPH_2_ from photosynthesis [34].

The 13 selected intracellular metabolites (Figure 2) were reduced across the UV-B time series indicating the reduction of cellular processes. A decrease in phe and tyr could be due to their role as precursors to many secondary metabolites such as aromatic nitrogen-containing alkaloids [13]. M-gly and shinorine are produced via a combination of the shikimate/pentose phosphate pathway which also involves the addition of gly and ser to form the final MAAs [9,35]. The reduction of these amino acids coincides with an increase in MAA levels (Figure 3B).

5-oxoproline and glu are involved in glutathione metabolism. 5-oxoproline reduction (*p* ≤ 0.01) could be due to its interconversion into glutamate which is further converted into the antioxidant glutathione [36]. Glu is also produced from the assimilation of nitrogen during nitrogen fixation which is reduced during UV-B exposure [34].

Pro has been studied in many UV-B experiments involving different photosynthetic organisms and its accumulation is thought to have a role in stress response by providing additional defence as a ROS scavenger and molecular chaperone [37,38,39]. Accumulation of proline has been observed in *Nostoc punctiforme* during 24 h of UV-A stress [18], in the model plant organism *Arabidopsis* after 24 h of UV-B treatment [40], and also in *C. fritschii* after 24 h of UV-B exposure within this study.

Overall less significant differences were observed during PAR only conditions (Figure 5) compared to PAR supplemented with UV-B (Figure 2).

### 3.2 Extracellular Metabolite Changes

The movement of metabolites and substrates between cells and their surrounding environment (or vice versa) can occur via passive and active uptake and efflux systems. Reactions can also occur on cell surface membranes and as transformations of media components [41]. Identification of extracellular metabolite uptake and release from cyanobacteria is, therefore, a complex process due to the high turnover rates of intracellular processes [25]. Extracellular metabolites can be released during stress and as by-products of intracellular reactions [19]. Sugars such as galactose, arabinose, lyxose and xylose are actively released during UV-B stress [5] as seen in this experiment (Figure 4).

7 metabolites from the identified biologically relevant pool were found in both intra- and extracellular metabolite samples (Figure S3). The fatty acid mystiric acid shows a similar pattern of reduced abundance with increasing length of UV-B in both samples (intracellular *p* ≤ 0.05; extracellular *p* ≤ 0.05). Ethanolamine, involved in glycerophospholipid metabolism, and 2-oxobutanoate, involved in amino acid biosynthesis show opposite patterns with intracellular levels decreasing (*p* ≤ 0.05) and extracellular levels increasing (ethanolamine, *p* ≤ 0.01, 2-oxobutanoate, *p* ≤ 0.001) (S1: Figure 3). Further detailed analysis using a combination of -omic techniques and the application of isotopic labelling such as ^13^C flux balance analysis would be required to better understand the uptake and release of extracellular metabolites and their possible use within cyanobacterial metabolite production [42].

## 4. Conclusions

In summary, an untargeted GC–MS workflow was used to evaluate intra- and extracellular metabolites under supplemented UV-B exposure (PAR + UV-B). Most significantly we found a reduction of intracellular metabolites such as the amino acids, tyr, phe, ser, gly and glu and the accumulation of pro, which to our knowledge has not been previously reported in *C. fritschii.* Compared to PAR only, intracellular metabolites showed less significant changes with amino acids tyr, phe and glu accumulation observed.

Although a time series analysis was conducted, this only represents a minuscule proportion of the true changes within the metabolome. This study is important to build on experimental data already available for cyanobacteria and other photosynthetic organisms exposed to UV-B. To understand the changes in primary metabolites and metabolic process with increasing length of UVR exposure to help further understand secondary metabolite production and adaptation of cyanobacteria to UV stress. Further studies are needed to understand and verify these processes within cyanobacteria to aid in the understanding of UV stress adaptation at the metabolite level.

## 5. Materials and Methods

### 5.1. Organism and Growth Conditions

The cyanobacterium *C. fritschii,* PCC 6912, was obtained from the Pasteur culture collection (PCC) and grown in autoclaved deionised water with filtered BG-11 growth medium (Sigma Aldrich). The strain was maintained in 50 mL BG-11 at a temperature of 27 ± 2 °C under continuous PAR illuminated at 15 µmol m^−2^ s^−1^ (measured using a PAR light sensor, Enviromonitors, West Sussex, UK). Experimental cultures were pre-grown in 300 mL BG-11 media under the same conditions with constant shaking at 80 rpm.

### 5.2. Experimental Setup

#### 5.2.1. Supplemented UV-B Experiment (PAR + UV-B)

After 6 days of pre-growth, triplicate experimental *C. fritschii* cultures were transferred into three Quartz Erlenmeyer flasks (H.Baumbach & CO.LTD, Suffolk, UK) at an optical density at 750 nm (OD_750nm_) of approx. 0.14 to allow even UV-B exposure. The cultures were exposed to a total of 48 h of UV-B radiation using a UVB broadband (290–315 nm, centered at 310 nm) fluorescent tube (Philips TL 20W/12 RS SLV/25, Proflamps, Eindhoven, The Netherlands) emitting 3 µmol m^−2^ s^−1^ of UV-B radiation (measured using a UVR light sensor, Enviromonitors, West Sussex, UK). The experiment was carried out under continuous PAR at 15 µmol m^−2^ s^−1^ and shaking at 100 rpm for even UVB exposure of cells. For time course analysis samples were collected at no UV-B (0 h), 2, 6, 12, 24 and 48 h for dry weight, pigment, MAA, and GC–MS analysis.

#### 5.2.2. PAR Only Experiment (PAR only)

*C. fritschii* cultures were pre-grown for 6 days prior to experimental analysis. After 6 days of pre-growth, triplicate experimental *C. fritschii* cultures (OD_750nm_ of approx. 0.13) were grown at a temperature of 27 ± 2 °C under continuous PAR illuminated at 15 µmol m^−2^ s^−1^ (measured using a PAR light sensor, Enviromonitors, West Sussex, UK) and continuous shaking at 100 rpm. For time course analysis samples were collected at 0, 2, 6, 12, 24 and 48 h for dry weight and GC–MS analysis.

### 5.3. Sample Harvest and Growth Analysis

Forty mL volumes of UV-B exposed (*n* = 3) and PAR only cultures (*n* = 3) were harvested at each time point by centrifugation at 4400 rpm for 20 min to produce a pellet and supernatant. The supernatants (40 mL) were collected and freeze-dried (Edwards, super modulyo) for 72 h. The remaining pellets were transferred into pre-weighed Eppendorf’s and freeze-dried for 24 h (Scanvac, CoolSafe^TM^, LaboGene^TM^, Vassingerød, Denmark) for dry weight measurements. Both pellets (PAR + UV-B and PAR only) and dried supernatant (PAR + UV-B only) were stored at −20 °C until analysis. OD was monitored using absorbance at 750 nm using a UV-visible spectrophotometer (Shimadzu, UV-2550, Kyoto, Japan).

### 5.4. GC–MS Analysis

#### 5.4.1. Sample Preparation

Polar and non-polar metabolites were extracted from UV-B exposed and PAR only dried cell pellets for GC–MS analysis. Briefly, approx. 0.5–1 mg (UV-B exposed) or 1.5–3 mg (PAR only) of dried biomass was re-suspended in 1 mL methanol:chloroform:water (2:2:1) and sonicated using a sonicator probe (Fisher Scientific, FB50) using 6 cycles of 20 s pulses at 40 Hz at 0 °C. After centrifugation (5 min at 12,000 rpm), 100 µL of each solvent layer (both methanolic and chloroform layers) were aliquoted into new Eppendorf’s and evaporate to dryness using a rotary vacuum concentrator (Eppendorf concentrator 5301).

Dried supernatant (PAR + UV-B) was re-suspended in 1 mL of methanol and centrifuged (4000 rpm, 5 min). Two hundred µL was aliquoted into new Eppendorf’s followed by evaporation to dryness and derivatisation as below.

#### 5.4.2. Sample Derivatisation

To each 200 μL dried sample, 30 μL of methoxyamine hydrochloride (23 mg) in pyridine (1.5 mL) was added and samples were heated at 70 °C for 45 min. Once cooled to room temperature, 50 μL of MSTFA+TMCS (Thermo Scientific^TM^, product no: TS-48915) was added and samples heated for an additional 90 min at 40 °C. Once cooled to room temperature, 10 μL of tetracosane dissolved in hexane (2 mg/mL) was added as an internal standard. Derivatised samples were transferred into auto-sample vials ready for analysis.

#### 5.4.3. GC–MS Analysis

Derivatised sample (1 µL) was loaded onto an Agilent HP-5MS capillary column (30 m × 0.25 mm × 0.25 um) in splitless mode at 250 °C. The GC was operated at a constant flow of 1 mL min^−1^ helium. The temperature program started at 60 °C for 1 min, followed by temperature ramping at 10 °C min^−1^ temp of to a final 180 °C, this was followed by a second temperature ramping at 4 °C min^−1^ to a final temp of 300 °C and held constant at 300 °C for 15 min. Data acquisition included a mass range of 50 to 650 and resulted in .D data files for analysis.

Chromatograms were deconvoluted using AMDIS (Automated Mass Spectral Deconvolution and Identification System) followed by alignments using the online portal SpectConnect, http://spectconnect.mit.edu/ [43], before identifying peaks using Golm metabolome database, www.gmd.mpimp-golm.mpg.de/, and the NIST 05 (National Institute of Standards and Technology) library [44]. MetaboAnalyst, www.metaboanalyst.ca/, was used for statistical analysis [45,46].

#### 5.4.4. GC–MS Data Processing

GC–MS data sets need deconvolution of co-eluting compounds, the freely available software AMDIS was used to process the chromatograms (.D) and produce .ELU files for alignment and conservative component identification using SpectConnect [43,47]. Settings for AMDIS were as followed [18]; Resolution = medium, sensitivity = medium, shape requirement = medium and component width was 10. The resulting .ELU files were uploaded to SpectConnect to produce matrices for further analysis and processing in Excel 2010 (Microsoft, USA). The integrated signal (IS) matrix generated was used for relative quantification of peaks. Triplicate missing data points (within time points) were assumed to be lower than the detection limit and replaced with half of the minimum integrated signal within each data set. Data normalisation was carried out using the peak area of the internal standard tetracosane and dry weight of each sample (for intracellular data only; approx. 0.5–1 mg of UV-B exposed cells and 1.5–3 mg of PAR only cells). All duplicate retention times were removed before further analysis.

#### 5.4.5. Identification

Identification of peaks was carried out in AMDIS by analysing each chromatogram using the Golm databases as a target library, followed by searching the NIST 05 library with a match factor of 60% or above. Reports were exported in .xls format from AMDIS with the first hit only included for further processing using Excel 2010 (Microsoft, Redmond, WA, USA). A true hit was considered when two or more biological replicates (within the same time-point) contained the peak. If none of the time points contained ‘true hits,’ the peaks were removed before further analysis. Metabolites reported belonged to level 2 (putatively annotated compounds) and level 4 (unknown compounds) identifications in accordance with the Metabolomics Standards Initiative [48].

#### 5.4.6. Statistical Analysis

MetaboAnalyst was used to statistically analyse the IS peak lists (in .csv format) of the time series data using the time-series/two-factor module. Multivariate analysis was carried out using each column as a different time point and each row representing a metabolite (data type = peak intensity table; study design = time-series only; data format = samples in columns) [46]. Missing data points were uploaded as blanks and replaced with half of the minimum integrated signal within each data set. Peaks were normalised to total sum of peaks, log-transformed and mean centered prior to statistical analysis. PCA, a one-way repeated ANOVA (*p* ≤ 0.05) and hierarchical heat map clustering was used to evaluate the data. A two-sample T-test with equal variance was also used as a univariate statistical tool to evaluate data comparing 0 h with each treatment time point (2, 6, 12, 24 and 48 h) as well as between each time points in Excel.

### 5.5. MAA analysis

#### 5.5.1. Sample Preparation

0.5–1 mg of UV-B exposed dried biomass was re-suspended in 100% HPLC grade methanol (1 mL) and left in the dark at 4 °C overnight (24 h). After centrifugation (5 min at 12000 rpm), the supernatant was removed and evaporated to dryness using a rotary vacuum concentrator. The dried extract was re-dissolved in 600 µL of deionised water and transferred to autosample vials for HPLC analysis [49].

#### 5.5.2. HPLC Analysis

HPLC analysis was performed using an Agilent 1100 system equipped with a binary pump (G1312A), an autosampler injector (ALS, G1313A), thermostatted column compartment (G1316A) and diode array detector (DAD, G1315A) connected via an interface module to a computer running ChemStation software. The stationary phase was an Alltima^TM^ Altech^TM^ C18, 4.6 × 150 mm, 5 µm column heated to 35 °C. The mobile phases consisted of; Eluent A: Water (0.01% TFA, *v/v*) and Eluent B: 70% methanol (0.054% TFA, *v/v*) with a gradient of; 99% A for 10 min, to 80% A over 5 min, to 1% A over 5 min, held for 3 min and increased to 99% A over 2 min. The samples were injected at a volume of 100 µL and MAA’s were monitored at wavelengths of; 310, 320 and 330 nm, absorption spectra between 200–400 nm were stored in each detected peak.

### 5.6. Pigment Analysis

#### Sample Preparation

To approx. 0.5–1 mg of dried biomass, 100% HPLC grade methanol (1 mL) was added and vortexed to re-suspend. Samples were sonicated under low light conditions using a sonicator probe for 6 cycles of 20 s pulses at 40 Hz at 0 °C. After centrifugation (5 min at 12,000 rpm), the supernatant was removed and absorbance spectra measured using a UV-visible spectrophotometer between 400–800 nm with 100% methanol as a blank. Carotenoid concentration was calculated using the equations as described in [50,51].

## Supplementary Materials

The following are available online at https://www.mdpi.com/2218-1989/9/4/74/s1, Figure S1: Hierarchical heatmap visualisation of the significant (A) intracellular and (B) extracellular peak intensities (*p ≤* 0.05) during UV-B exposure using a one-way repeated measure ANOVA in MetaboAnalyst. Data is arranged in triplicate with increasing length of UV-B exposure from left to right (0–48 h). S1 = replicate 1, S2 = replicate 2, S3 = replicate 3; Figure S2: Dry weight measurements of *C. fritschii* cultures during 48 h of supplemented UV-B exposure. All values are the mean of three biological replicates ± standard error; Figure S3: Time-series metabolomics data of *C. fritschii* during 48 h of UV-B exposure showing primary metabolites found in both intra- and extracellular during GC–MS analysis. (A) Metabolites showing statistically significant (*p* ≤ 0.05) changes over time in intra- and extracellular data; (B) metabolites showing statistically significant (*p* ≤ 0.05) changes over time in intracellular samples only. Statistical significance was measured using a two-sample T-test comparing control (0 h) to each treatment time point (2, 6, 12, 24 and 48 h) and between treatment time points, * = 0.05 ≥ *p* ≥ 0.01, ** = 0.01≥ *p* ≥ 0.001 and *** = *p* ≤ 0.001; Table S1: Intracellular compounds detected during UV-B exposure using GC–MS including; Retention time (RT), name, class, model (*m/z*), normalised mean abundance (*n* = 3) ± standard error (SE). T-test and ANOVA results showing statistical significant compounds, * = 0.05 ≥ *p* ≥ 0.01, ** = 0.01 ≥ *p* ≥ 0.001, *** = *p* ≤ 0.001; Table S2: Extracellular compounds detected during UV-B exposure using GC–MS including; Retention time (RT), name, class, model (*m/z*), normalised mean abundance (*n* = 3) ± standard error (SE). T-test and ANOVA results showing statistical significant compounds, * = 0.05 ≥ *p* ≥0.01, ** = 0.01 ≥ *p* ≥ 0.001, *** = *p* ≤ 0.001; Table S3: Biologically relevant metabolites detected during PAR + UV-B (intra- and extracellular metabolites) and PAR only (intracellular metabolites), including possible biosynthetic pathways; Table S4: Intracellular compounds detected during PAR only exposure using GC–MS including; Retention time (RT), name, class, model (*m/z*), normalised mean abundance (*n* = 3) ± standard error (SE). T-test and ANOVA results showing statistical significant compounds, * = 0.05 ≥ *p* ≥ 0.01, ** = 0.01 ≥ *p* ≥ 0.001, *** = *p* ≤ 0.001.
