# Supplementary material for: Intracellular and Extracellular Metabolites from the Cyanobacterium Chlorogloeopsis fritschii, PCC 6912, During 48 Hours of UV-B Exposure
Source: Metabolites. 2019 Apr 16;9(4):74. doi: 10.3390/metabo9040074 (PMC6523833; doi:10.3390/metabo9040074)
Supplement: Supplementary file 1 [file metabolites-09-00074-s001.zip › Supplementary material S1_Figures.pdf]

metabolites

Supplementary Material

Intracellular and Extracellular Metabolites from the Cyanobacterium *Chlorogloeopsis fritschii*, PCC 6912, During 48 Hours of UV-B Exposure

Bethan Kultschar [1,*], Ed Dudley [2], Steve Wilson [3] and Carole A. Llewellyn [1,*]

[1] Department of Biosciences, Swansea University, Singleton Park, Swansea, SA2 8PP, UK
[2] Swansea University Medical School, Swansea University, Singleton Park, Swansea, SA2 8PP, UK; E.Dudley@swansea.ac.uk
[3] Unilever Corporate Research, Colworth Park, Sharnbrook, Bedfordshire, MK44 1LQ, UK Steve.Wilson@Unilever.com
* Correspondence: 878620@swansea.ac.uk (B.K.), C.A.Llewellyn@swansea.ac.uk (C.A.L.); Tel.: +44-1792-606168 (C.A.L.)

Received: 12 March 2019; Accepted: 13 April 2019; Published: 16 April 2019

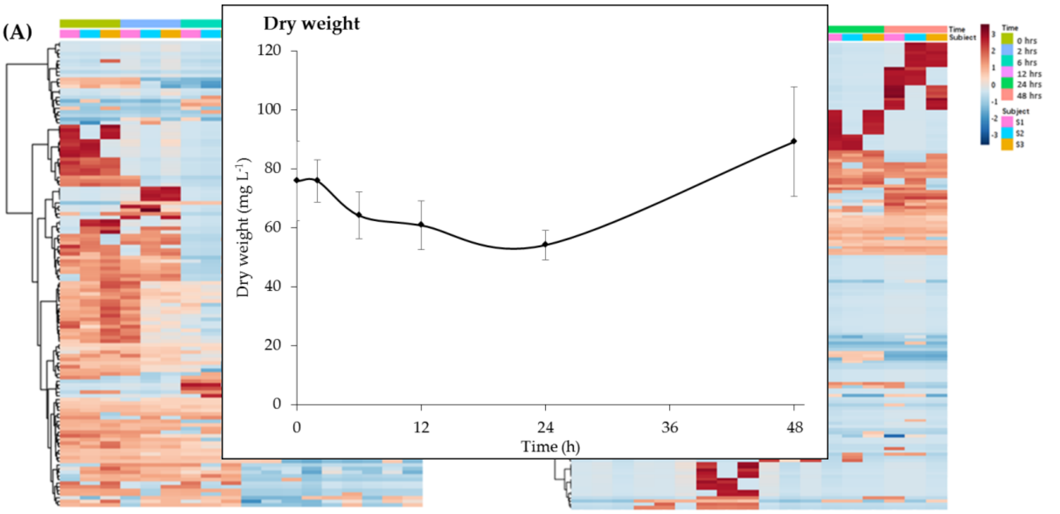

Figure S1: Hierarchical heatmap visualisation of the significant **(A)** intracellular and (**B)** extracellular peak intensities (*p* ≤0.05*)* during UV-B exposure using a one-way repeated measure ANOVA in MetaboAnalyst. Data is arranged in triplicate with increasing length of UV-B exposure from left to right (0–48 h). S1 = replicate 1, S2 = replicate 2, S3 = replicate 3.

Figure S2: Dry weight measurements of *C. fritschii* cultures during 48 h of supplemented UV-B exposure. All values are the mean of three biological replicates ± standard error.

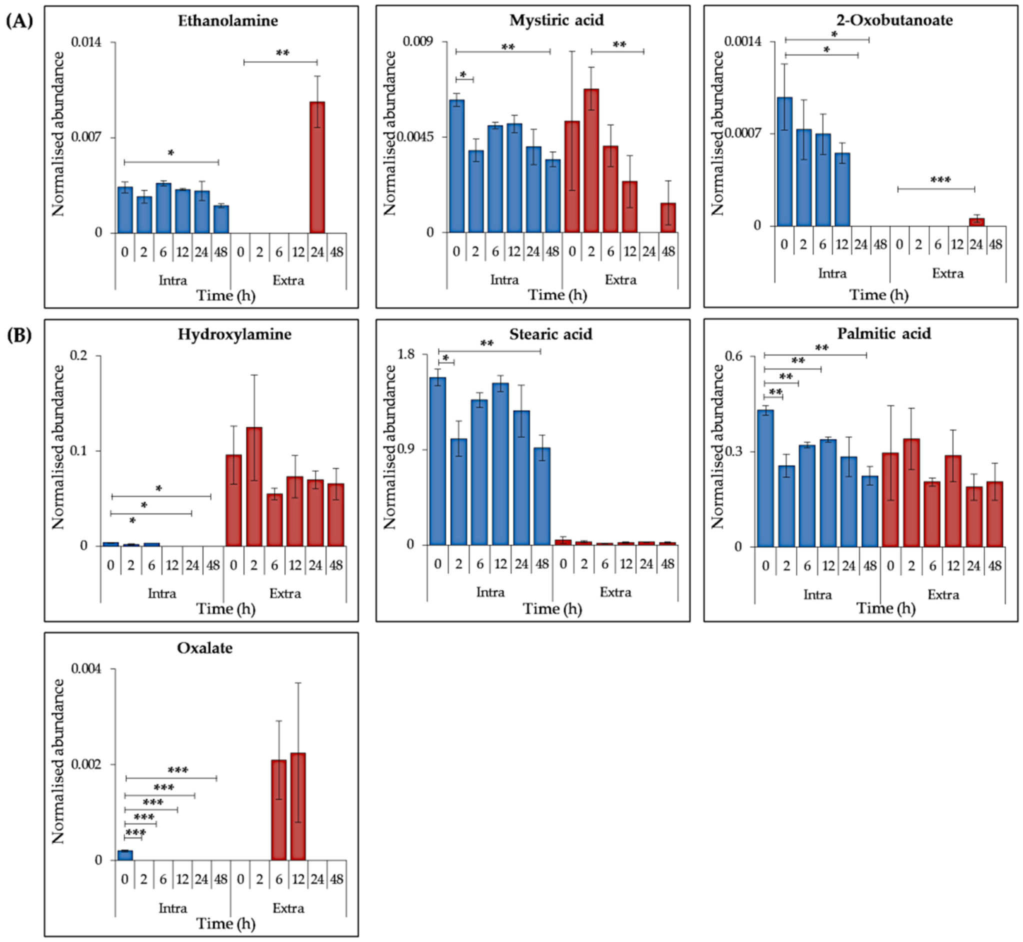

Figure S3: Time-series metabolomics data of *C. fritschii* during 48 h of UV-B exposure showing primary metabolites found in both intra- and extracellular during GC-MS analysis. **(A)** Metabolites showing statistically significant ($p \leq 0.05$) changes over time in intra- and extracellular data; **(B)** metabolites showing statistically significant ($p \leq 0.05$) changes over time in intracellular samples only. Statistical significance was measured using a two-sample T-test comparing control (0 h) to each treatment time point (2, 6, 12, 24 and 48 h) and between treatment time points, * = $0.05 \geq p \geq 0.01$, ** = $0.01 \geq p \geq 0.001$ and *** = $p \leq 0.001$.
